# Supplementary material for: fLPS 2.0: rapid annotation of compositionally-biased regions in biological sequences
Source: PeerJ. 2021 Oct 28;9:e12363. doi: 10.7717/peerj.12363 (PMC8557692; doi:10.7717/peerj.12363)
Supplement: Supplemental Information 1 — UP000005640_9606.wPFdomains.ALL.PF00076.PRD Bin ’x’ holds all values in the range x−5.0 to x. [file peerj-09-12363-s001.pdf]

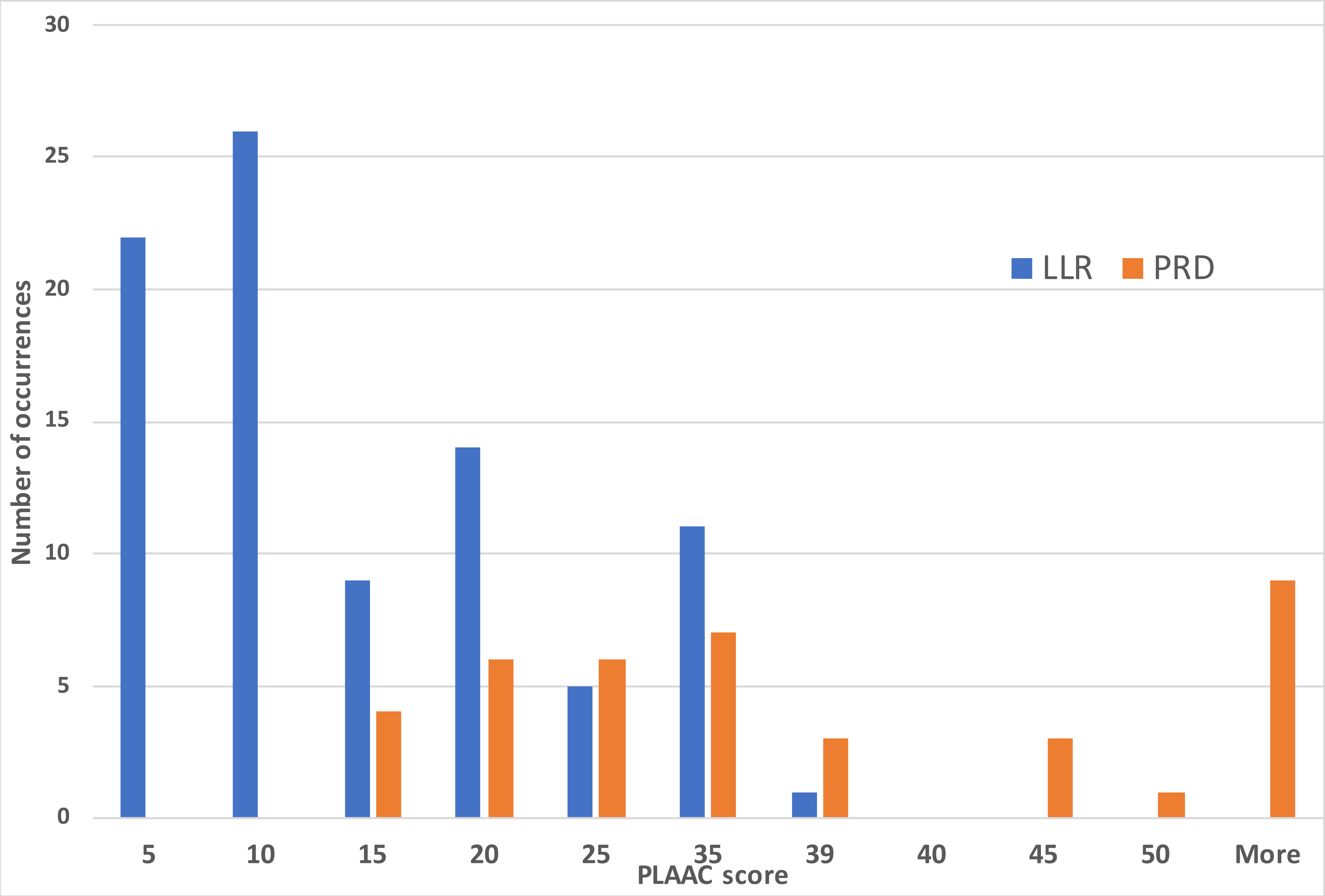


**Supplementary Figure 1: Annotations of prion-like domains are binned in histograms according to their PLAAC score (either the LLR score or PRD score). Bin 'x' holds all values in the range x-5.0 to x.**
